# Advancing Dementia Care: Memory Aid Technology and Data‐Driven Insights for Autonomy at Home

**DOI:** 10.1002/alz.084275

**Published:** 2025-01-09

**Authors:** Alyssia A Sanchez, Alex Mihailidis

**Affiliations:** ^1^ University of Toronto, Pickering, ON Canada; ^2^ University of Toronto, Toronto, ON Canada

## Abstract

**Background:**

Assistive technology (AT) plays a crucial role in empowering people living with dementia (PLWD) to perform tasks independently, enhancing their autonomy and dignity. To build on this foundation, our proposal introduces a home‐based reminder system designed to further support PLWD in their daily lives.

**Hypothesis:**

Memory aid technology, in particular reminder systems, can be developed to prospectively provide PLWD with autonomy and independence, to alleviate responsibilities and time commitments of caregivers and clinicians, and to enable remote behavioral monitoring.

**Method:**

Our project is grounded in existing research and focuses on the development and implementation of an innovative home‐based reminder system. This system comprises electronic reminder units and a central base station, enabling remote behavior monitoring and communication. Caregivers can transmit reminders through a mobile application, and the reminder units display these reminders and detect when they’re acknowledged by PLWD, allowing for the collection and analysis of behavioral data. Machine learning models are employed to understand daily schedules and identify deviations from routines. We engage dyads of PLWD and family caregivers through interviews and usability testing to refine the system.

**Results:**

Preliminary stages of development involve system development, data simulation with approximately 1000 days of reminder usage, and interviews and useability demonstrations with 8 PLWD‐caregiver dyads. These efforts indicate the potential of memory aid technology to enhance communication between PLWD and caregivers, becoming a valuable aspect of daily routines. The reminder system can evolve to monitor and analyze behavior, providing crucial insights into the daily lives of PLWD and enabling timely support and intervention when needed.

**Conclusion:**

Through the development and evaluation of this innovative reminder system, our aim is to improve the lives of PLWD, augment their autonomy, and assist caregivers in delivering effective and personalized dementia care. The system’s potential to enhance communication and provide behavioral insights aligns with the criteria for dementia care practice proposals, offering a promising avenue for advancing care practices and contributing to broader improvements in dementia care within our aging society.

